# Screening for latent and active tuberculosis infection in the elderly at admission to residential care homes: A cost-effectiveness analysis in an intermediate disease burden area

**DOI:** 10.1371/journal.pone.0189531

**Published:** 2018-01-02

**Authors:** Jun Li, Benjamin H. K. Yip, Chichiu Leung, Wankyo Chung, Kin On Kwok, Emily Y. Y. Chan, Engkiong Yeoh, Puihong Chung

**Affiliations:** 1 Jockey School of Public Health and Primary Care, Chinese University of Hong Kong, Hong Kong, China; 2 Tuberculosis and Chest Service, Department of Health, Hong Kong, China; 3 Department of Public Health Science, Graduate School of Public Health, Seoul National University, Seoul, South Korea; Chinese Academy of Medical Sciences and Peking Union Medical College, CHINA

## Abstract

**Background:**

Tuberculosis (TB) in the elderly remains a challenge in intermediate disease burden areas like Hong Kong. Given a higher TB burden in the elderly and limited impact of current case-finding strategy by patient-initiated pathway, proactive screening approaches for the high-risk group could be optimal and increasingly need targeted economic evaluations. In this study, we examined whether and under what circumstance the screening strategies are cost-effective compared with no screening strategy for the elderly at admission to residential care homes.

**Methods:**

A decision analytic process based on Markov model was adopted to evaluate the cost-effectiveness of four strategies: (i) no screening, (ii) TB screening (CXR) and (iii) TB screening (Xpert) represent screening for TB in symptomatic elderly by chest X-ray and Xpert® MTB/RIF respectively, and (iv) LTBI/TB screening represents screening for latent and active TB infection by QuantiFERON®-TB Gold In-Tube and chest X-ray. The target population was a hypothetical cohort of 65-year-old people, using a health service provider perspective and a time horizon of 20 years. The outcomes were direct medical costs, life-years and quality-adjusted life-years (QALYs) measured by incremental cost-effectiveness ratio (ICER).

**Results:**

In the base-case analysis, no screening was the most cost-saving; TB screening (CXR) was dominated by TB screening (Xpert); LTBI/TB screening resulted in more life-years and QALYs accrued. The ICERs of LTBI/TB screening were US$19,712 and US$29,951 per QALY gained compared with no screening and TB screening (Xpert), respectively. At the willingness-to-pay threshold of US$50,000 per QALY gained, LTBI/TB screening was the most cost-effective when the probability of annual LTBI reactivation was greater than 0.155% and acceptability of LTBI/TB screening was greater than 38%. In 1,000 iterations of Monte Carlo simulation, the probabilities of no screening, TB screening (CXR), TB screening (Xpert), and LTBI/TB screening to be cost-effective were 0, 1.3%, 20.1%, and 78.6% respectively.

**Conclusions:**

Screening for latent and active TB infection in Hong Kong elderly people at admission to residential care homes appears to be highly effective and cost-effective. The key findings may be the next key factor to bring down TB endemic in the elderly population among intermediate TB burden areas.

## Introduction

Tuberculosis (TB) continues to be one of the world’s biggest health threats under the era of population ageing [1]. In Hong Kong, a developed city with intermediate disease burden, the TB notification rates had shown an overall downward trend from a peak of 697/100,000 in 1952 to 114/100,000 in 1990 and became rather stagnant in the following years [2]. Affected by ageing of population and TB endemic, the TB notification rates in people aged 65 years and above increased in the 1990s and remained four times that in younger people in 2014 (181 vs. 45/100,000) [2]. TB incidence and mortality are known to increase with age, especially in long-term care facilities for the elderly where densely populated individuals with diverse comorbidities have a greater risk [3, 4].

The residential care homes for the elderly (RCHEs) in Hong Kong provide different levels of care principally for people aged 65 years and above. In 2016, around 930 non-private and private RCHEs provided over 74,000 residential placements and care for approximately 7% of the elderly population [5]. In such settings, previous studies demonstrated a high prevalence of latent TB infection (LTBI) ranging from 43.8% to 69.6% [6–9]. The prevalence of active TB ranged from 0.7% to 2.6%, much higher than the annual TB notification rate [8–10]. In addition to the heavy disease burden, endogenous reactivation increasingly accounted for most elderly TB cases compared with that from primary infection and exogenous reinfection [11, 12]. However, current case-finding strategy by patient-initiated pathway (also labeled as “passive case-finding strategy”) had limited impact on preventing the reactivation of LTBI [12, 13]. In the patient-initiated pathway, TB program does not actively detect new cases outside healthcare facilities but concentrates on delivering high-quality diagnosis and treatment services to those who present suspicious TB symptoms (herein termed “symptoms”) and approach the healthcare facilities [14]. Atypical symptoms presentation in the elderly, lack of timely and accurate diagnostic tools, and low awareness would lead to a longer delay in diagnosis and treatment in RCHEs and subsequently result in further transmission and death due to TB [15, 16].

In order to eliminate the disease, early detection of LTBI and TB through screening pathway along with appropriate treatment in high-risk groups is highly recommended by the World Health Organization (WHO) guidelines and endorsed by the End TB Strategy [17–19]. In Japan, older people are prioritized for TB screening program in facilities for the aged [20]. In the USA, screening for LTBI and TB is recommended to older people being newly admitted to long-term care facilities [21]. In Hong Kong, however, no such targeted programs and interventions were available, partly due to the absence of cost-effectiveness evidence [22, 23].

Meanwhile, rapid diagnostic tools were emerging in the past decade. Interferon-gamma release assays (IGRAs), such as QuantiFERON®-TB Gold In-Tube (QFT-GIT) [Cellestis Ltd, Carnegie, Victoria, Australia], are alternatives for LTBI diagnosis with a higher accuracy compared with tuberculin skin test (TST) [15, 22]. It is more convenient and less affected by advanced age, Bacillus Calmette-Guerin (BCG), and booster phenomenon [15, 22]. Compared with Chest X-Ray (CXR) and/or sputum smear microscopy as the initial test in TB screening, Xpert® MTB/RIF (Xpert) [Cepheid Inc, Sunnyvale, CA, USA] is a more rapid molecular diagnostic tool with a higher sensitivity in smear-positive samples and negative predictive value [24, 25]. It can diagnose TB and drug resistance to rifampin within two hours, which will provide accurate and prompt evidence for an initial treatment.

Field implementation of IGRAs and Xpert in screening also requires economic evaluation under specific epidemiologic situation. To date, few cost-effectiveness studies of screening by the new diagnostic tools concentrated on the aged population, particularly in the areas with intermediate TB burden [26–30]. In order to bridge the policy and research gap, we conducted a cost-effectiveness study to examine whether and under what circumstance screening strategies are cost-effective compared with no screening strategy for the elderly at admission to RCHEs in Hong Kong.

## Method

A decision analytic process based on Markov model was adopted to evaluate the cost-effectiveness of three screening strategies compared with no screening strategy. The model adopted health service provider perspective and a time horizon of 20 years in line with the End TB Strategy targets (90% reduction in TB incidence and 95% reduction in TB deaths between 2015 and 2035) [17]. The target population was a hypothetical cohort of the 65-year-old elderly population at admission to RCHEs. No ethics approval was required for this modelling study. Model development and analysis utilized TreeAge Software (TreeAge Software Inc., Williamstown, MA, USA).

### Screening strategies

**Fig 1:**
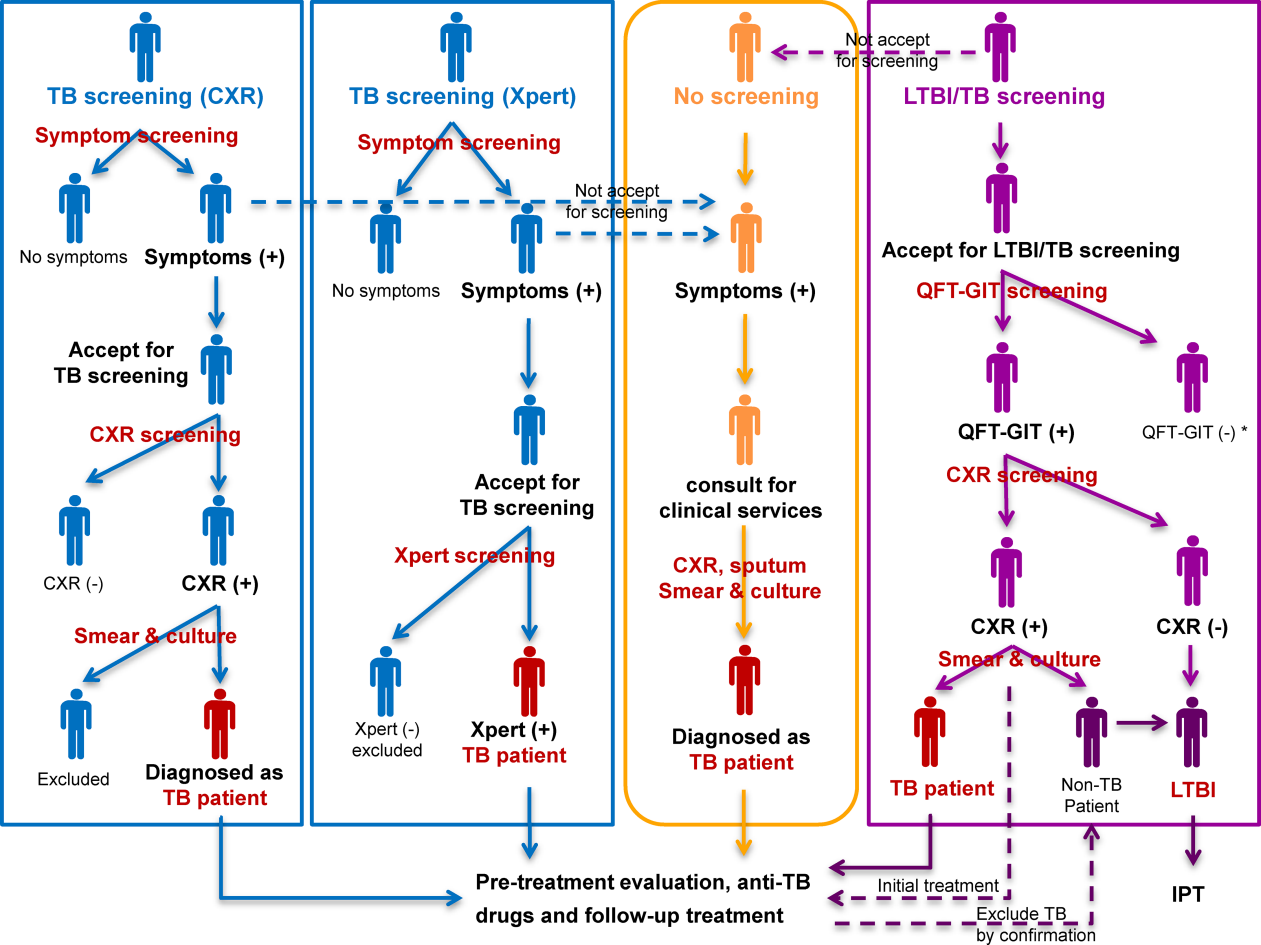
Simplified algorithms of no screening and three screening strategies. *Those with QFT-GIT negative results follow no screening strategy.

Three screening strategies were selected and structured in the model according to the latest evidence from the WHO guidelines, previous cost-effectiveness studies of TB screening, and TB epidemiological studies in Hong Kong RCHEs [6–9, 18, 19, 31–33]. No screening strategy represents the current TB case-finding strategy principally by patient-initiated pathway in Hong Kong [10]. All strategies are presented in Fig 1.

1. No screening. People who present symptoms and approach the healthcare facilities are examined by primary diagnostic tools, including CXR, sputum smear microscopy, and culture test. Pre-treatment evaluation, anti-TB drugs, and follow-up treatment are provided to TB patients. No further follow-up is needed for those without TB.
2. TB screening (CXR). It represents the screening strategy for TB by the initial diagnostic tool of CXR in TB suspects. All elderly population is investigated of symptoms at admission to RCHEs, where symptomatic ones are promoted to CXR test. For those do not accept the screening, the patient-initiated pathway in no screening strategy is alternatively considered. People with an abnormal presentation in CXR will be further examined by smear sputum microscopy and culture test. Pre-treatment evaluation, anti-TB drugs, and follow-up treatment are provided to people who are diagnosed with TB. No further follow-up is needed for those without TB.
3. TB screening (Xpert). It represents the screening strategy for TB by the diagnostic tool of Xpert in TB suspects. All elderly population is initially investigated of symptoms at admission to RCHEs, where symptomatic ones are promoted to Xpert test. For those do not accept the screening, the patient-initiated pathway is alternatively considered. People with positive results of Xpert are directly diagnosed with TB patients and provided with pre-treatment evaluation, anti-TB drugs, and follow-up treatment. If the Xpert result is negative, the individual has no further follow-up.
4. LTBI/TB screening. It represents the screening strategy for LTBI and TB by the initial diagnostic tools of IGRAs and CXR. As one available product of IGRAs in Hong Kong, QFT-GIT is adopted for screening LTBI in the model. All elderly population is promoted to be first screened by QFT-GIT at admission to RCHEs. For those do not accept screening, the patient-initiated pathway is alternatively considered. If the QFT-GIT result is positive, CXR is performed to further distinguish active TB patient from LTBI. Those with positive QFT-GIT results and abnormal CXR presentations are initially diagnosed with TB patients and provided with pre-treatment evaluation and TB treatment. The confirmation is made by following smear sputum microscopy and culture test. Those with positive QFT-GIT results and normal CXR presentation (and those with positive QFT-GIT results and exclusive of TB) are diagnosed with LTBI and provided with isoniazid preventive therapy (IPT). If the result of QFT-GIT is negative, the individual has no further follow-up but may consider the patient-initiated pathway later when they develop the symptoms.

### Model design

**Fig 2:**
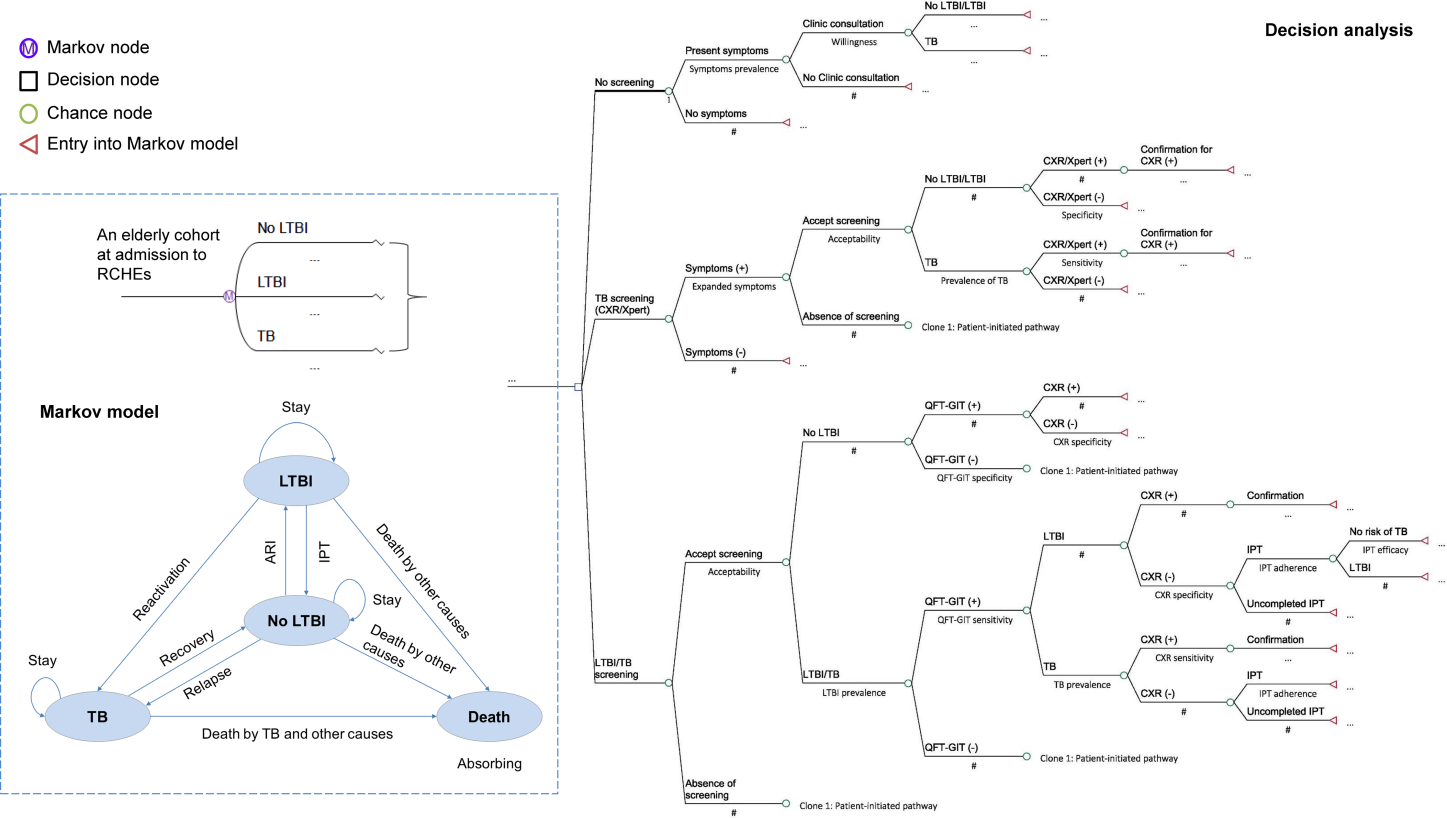
Simplified decision analytic process based on Markov model. Model states in three screening strategies for subsequent years where screenings do not occur are identical to no screening strategy (Clone 1: patient-initiated pathway). In decision analysis, decision node represents the selection of alternative strategies, and chance node represents the selection of probabilities. Markov node indicates the start of Markov model and represents the selection of health states in a number of Markov cycles.

A simplified decision analytic process based on Markov model is presented in Fig 2. Within the model, each individual at admission to RCHEs is assigned one of three initial health states: No LTBI, LTBI, and TB. In each one-year Markov cycle, the individual is either stay or transit to another health state until death. The model was designed to capture the essence of TB natural history and impacts of early detection and treatment in strategies by simulating a cohort of aged population through the transition of health states within 20 Markov cycles.

### Model probabilities and assumptions

Table 1 provides all the probability parameters used in the model. In the Markov model, the proportions of the initial health state of LTBI and TB were estimated from previous studies in Hong Kong RCHEs [6–9]. All the elderly in no LTBI state have an annual risk of infection (ARI) estimated by the standard formula ARI = 1- (1-Infection prevalence)^1/Age^, assuming a constant rate of exponential decline in the proportion of uninfected populations [34]. In Hong Kong, it was assumed that recent transmission only accounted for 15–20% in registered TB cases, while LTBI reactivation accounted for the vast majority of elderly TB patients [11, 12]. The probability of annual LTBI reactivation in the elderly was synthesized from studies in Hong Kong and high-income countries [11, 12, 32, 33, 35]. Those with LTBI have little risk of reactivation if efficacious IPT is completed [36–40], but might be re-infected by ARI. The probabilities of recovery by successful TB treatment, relapse, and mortality were extracted from annual TB report and previous studies in Hong Kong [2, 41–43]. Considering that death in the elderly significantly increases with age, we adopted age-specific mortality rates due to TB and other causes in 5-year intervals [2, 44]. Previous studies suggested that active screening could reduce delay in diagnosis by 50%, and therefore transmission by 30% [45]; failure to receive early anti-TB treatment had a 1.8 hazard ratio of death [46]. In the model, we decreased the coefficient of ARI by a factor of 0.7 and the coefficients of TB mortality by dividing 1.8 in the initial Markov cycle where screening occurs to reflect the effects of early detection and treatment.

**Table 1:** Model parameters of probabilities.

Model inputs	Baseline value	Range of sensitivity analysis	Reference
Prevalence of LTBI	0.57	0.438–0.696	[6–9]
Prevalence of TB	0.012	0.006–0.026	[8, 9]
Annual risk of TB infection	0.013	0.0095–0.0182	[6–9]
Probability of annual LTBI reactivation	0.0025	0.0004–0.006	[11, 12, 32, 33, 35]
Probability of TB relapse after treatment	0.012	0.008–0.016	[2, 43]
Probability of successful treatment among TB patients	0.766	0.725–0.819	[41–43]
Probability of TB death in treated patients			
age 65–69 years old	0.028	0.01–0.046	[2]
age 70–74 years old	0.069	0.042–0.097	[2]
age 75–79 years old	0.068	0.043–0.093	[2]
Age 80–84 years old	0.084	0.057–0.111	[2]
Probability of TB death in untreated smear positive (negative) patients	0.113 (0.022)	0.073–0.178 (0.01–0.035)	[51]
Probability of death by other causes			
age 65–69 years old	0.009	0.0087–0.0097	[2, 44]
age 70–74 years old	0.017	0.0158–0.0173	[2, 44]
age 75–79 years old	0.027	0.0257–0.0276	[2, 44]
age 80–84 years old	0.047	0.0458–0.0488	[2, 44]
Proportion of smear positive in annual registered TB patients	0.376	0.352–0.401	[2]
Probability of cough in any duration, hemoptysis, and/or weight loss in elderly population	0.2	0.139–0.337	[8, 9, 48, 49]
Probability of cough ≥3 weeks and/or hemoptysis in elderly population	0.0327	0.031–0.034	[48]
Probability of cough in any duration, hemoptysis, and/or weight loss in elderly TB patients	0.6	0.5–0.7	[2, 47, 48]
Probability of cough ≥3 weeks and/or hemoptysis in elderly TB patients	0.36	0.277–0.441	[48]
Probability of willingness to approach healthcare facilities when cough ≥3 weeks and/or hemoptysis	0.7	0.5–0.9	[52]
Sensitivity of CXR	0.7	0.59–0.9	[18, 53–55]
Specificity of CXR	0.6	0.52–0.9	[18, 53–55]
Sensitivity of Xpert for smear positive TB	0.98	0.95–1	[24, 25]
Sensitivity of Xpert for smear negative TB	0.72	0.5–0.9	[24, 25]
Specificity of Xpert	0.99	0.95–1	[24, 25]
Sensitivity of QFT-GIT	0.84	0.81–0.87	[56]
Specificity of QFT-GIT	0.99	0.98–1	[56]
Acceptability of screening for TB	0.83	0.72–0.95	[18]
Acceptability of screening for LTBI/TB	0.6	0.35–0.85	[7, 8]
Adherence rate of IPT	0.8	0.5–0.9	[36,57–60]
Efficacy of IPT	0.85	0.6–0.9	[36–40]
Probability of isoniazid-induced hepatotoxicity	0.017	0.003–0.036	[38–40, 50, 61, 62]
Probability of hepatotoxicity in TB treatment	0.086	0.073–0.099	[2]

In the decision analytic process, the sensitivity and specificity of diagnostic tools, as well as the personal preference and performance (e.g., willingness, acceptability, and adherence) related parameters were synthesized from a number of previous studies including systematic review and meta-analysis if available. Other epidemiological parameters were extracted from Hong Kong annual TB report. The symptoms in the patient-initiated pathway were defined as cough more than 3 weeks and/or hemoptysis according to the TB manual [10]. In an attempt to maximize the effects of screening, we expanded the symptoms to cough in any duration, hemoptysis, and/or weight loss in TB screening strategy [2, 8, 9, 47–49]. In the diagnostic process of TB, smear-negative TB patients are followed by diagnostic antibiotic trial using first-line empirical treatment for pneumonia [10]. Before the treatment of LTBI and TB, blood tests for liver function, renal function, hepatitis B surface antigen (HBsAg), and human immunodeficiency virus (HIV) antibody are conducted for pre-treatment evaluation [10]. During the six months follow-up treatment, consultations for doctors, regular monitoring and tests are necessary [10]. Directly observed therapy (DOT) in TB patients is conducted by public health nurses, while drug-taken for LTBI treatment was assumed as self-administered [10]. To largely control the uncommon death due to serious side effects including (but not limited to) hepatotoxicity, we adopted pre-test for liver function, close monitoring during treatment, and hospitalization for those serious cases [10, 50].

### Cost estimation

From the health service provider perspective, only direct medical costs were considered in the model (Table 2). In Hong Kong, public health services for local residents are subsidized by the government. Nonlocal residents are charged in public hospitals by Hong Kong Hospital Authority based on the rate published in the Government Gazette [63]. We adopted the Hospital Authority charges as cost parameters for the majority of clinical services. As the new diagnostic tools, costs per test of Xpert and QFT-GIT were estimated from consumable and manpower costs according to a previous study and quotation of the QIAGEN company in Hong Kong [46]. For the costs of manpower resource in screening, diagnosis, and treatment, average hourly incomes of physician and nurse were used at an advanced level (experience of more than 5 years) and calculated according to the public data from the government and annual census [64, 65]. We estimated the average time of health service per case by interviewing doctors and nurses through a field investigation in Yuen Chau Kok Chest Clinic in Hong Kong.

**Table 2:** Model parameters of costs (USD, 1 USD = 7.8 HKD).

Cost	Baseline value	Range	Reference
**Clinical services**			
CXR	11	8.8–13.2	[63]
Sputum microscopy test	7.5	6–9	[63]
Culture test	45	36–54	[63]
Xpert test	128	102–154	[46]
QFT-GIT test	70	56–84	Estimation
IPT (6 months)	60	48–72	[63]
Diagnosis antibiotic trial	340	272–408	[63]
Pre-treatment evaluation*	110	88–132	[63]
First-line drugs for TB (6 months)	162	130–195	[63]
TB follow-up treatment (6 months)**	293.5	235–352	[63]
TB hospitalizations per day	600	480–720	[63]
The average days of hospitalization	15	10–20	[66]
**Manpower resource**			
Average physician income per hour	72	58–86	[64, 65]
Average nurse income per hour	40	32–48	[64, 65]
Average time for health service per case (minutes)			Investigation
TB consultant and diagnosis (physician)	40	32–48	
Case management and education (nurse)	60	48–72	
Promotion for screening and education (nurse)	30	24–36	
Symptom screening and sputum collection (nurse)	30	24–36	
Blood sample collection (nurse)	30	24–36	
DOT for TB treatment by three times per week (nurse)	10×72	576–864	
Month consultant during follow-up for LTBI/TB treatment (physician)	30×6	144–216	

* Pre-treatment evaluation includes blood tests for liver function, renal function, HBsAg, and HIV antibody.

** TB follow-up treatment includes CXR for two times, sputum microscopy, culture and Liver function test on average for three times.

### Outcome for effectiveness

By early detection and treatment, the overall goal of screening strategies was to prevent more TB cases and deaths by reducing the related risks, and eventually to protect public health and promote life expectancy and quality. Measuring effectiveness outcomes requires a comprehensive consideration of the whole-process effects. In addition, the outcomes should be comparable with previous related studies and other public health programs to facilitate the decision making in resource allocation [67]. Thus, life-years (LYs) and quality-adjusted life-years (QALYs) gained were selected as the effectiveness outcomes in comparison of strategies. QALYs estimations in TB patients were based on the LYs and utility weights with or without treatment and drug-related hepatotoxicity [68, 69] (Table 3).

**Table 3:** Model parameters of utility.

Model inputs	Baseline value	Range of sensitivity analysis	Reference
Complete health	1		
Treated active TB disease	0.85	0.7–0.9	[68, 69]
Untreated active TB disease	0.7	0.5–0.9	[68, 69]
Drug-related hepatotoxicity	0.8	0.7–0.95	[68]
Death	0		

### Cost-effectiveness analysis and sensitivity analysis

The cost-effectiveness analysis synthesized cost and effectiveness by calculating incremental cost-effectiveness ratio (ICER). Compared with one of the competing alternatives, a strategy is absolutely dominated when it is less effective and more costly and classified as extended dominance when it is less effective and has a higher ICER [67]. After excluding the dominance, the most cost-effective strategy was defined as the one with highest ICER under the willingness-to-pay (WTP) threshold, which was US$50,000 per QALY gained in this study [70]. The costs and effectiveness outcomes were discounted at an annual rate of 5% and adjusted by half-cycle correction [67, 71].

To examine model uncertainty on robustness of results, deterministic sensitivity analysis was performed within the range of each parameter (Tables 1–3). The minimum and maximum values were obtained from literature review and 95% confidence intervals of data-derived distributions if available. A range of variation by ± 20% of the base-case value was adopted for the cost parameters. Probabilistic sensitivity analysis was also conducted to simultaneously explore the uncertainty involved in key parameters. A Monte-Carlo simulation was conducted with 1,000 iterations where the key parameters were randomly drawn from probabilistic distributions (S1 Table). The WTP thresholds other than US$50,000 per QALY gained was also tested for selecting an optimal strategy.

## Results

### Base-case analysis

Table 4 presents results of the base-case analysis. Costs increased from no screening to TB screening (Xpert), TB screening (CXR), and LTBI/TB screening. All three screening strategies gained more LYs and QALYs compared with no screening; LTBI/TB screening was the most effective strategy. TB screening (CXR) cost more but gained less effectiveness compared with TB screening (Xpert), and was accordingly dominated. Under the WTP threshold of US$50,000 per QALY gained, LTBI/TB screening was the most cost-effective strategy.

**Table 4:** Cost-effectiveness of four strategies.

Strategy	No screening	TB screening (Xpert)	TB screening (CXR)	LTBI/TB screening
Cost (US$) per person	121	162	168	430
Incremental cost (US$)	Reference	41	47	309
	-	Reference	6	268
LYs accrued per person	11.1907	11.1952	11.1942	11.2003
Incremental LYs gained	Reference	0.0045	0.0035	0.0096
	-	Reference	-0.001	0.0051
QALYs accrued per person	11.1634	11.1702	11.1687	11.1792
Incremental QALYs gained	Reference	0.0068	0.0053	0.0158
	-	Reference	-0.0015	0.009
ICER (US$/LYs)	Reference	9,076	13,257	32,150
	-	Reference	Dominance	52,613
ICER (US$/QALYs)	Reference	6,094	8,935	19,712
	-	Reference	Dominance	29,951

### Deterministic sensitivity analysis

Table 5 provides the ranges of various WTP thresholds and selected parameters where the optimal strategy may vary from one to another. In the base-case analysis, LTBI/TB screening was the most cost-effective option when the WTP threshold was greater than US$29,951 per QALY gained, while the optimal strategy would switch to TB screening (Xpert) between the range of US$6,094 and US$29,951 per QALY gained and even to no screening if less was expected to be paid. In one-way sensitivity analysis, when the discount rate was not considered, LTBI/TB screening was more likely to be the optimal strategy as the lower limit of the WTP range decreased.

**Table 5:** Optimal strategy according to the willingness-to-pay threshold (US$/QALYs) in one way sensitivity analysis.

Scenario/Probability parameter	Value	Optimal strategy when WTP =			
		No screening	TB screening (Xpert)	TB screening (CXR)	LTBI/TB screening
Basic case		[0, 6,094)	[6,094, 29,951)	Dominance	[29,951, +∞)
Discount rate	0	[0, 4,084)	[4,084, 16,576)	Dominance	[16,576, +∞)
Probability of annual LTBI reactivation	0.0004	[0, 6,856)	[6,856, 254,461)	Dominance	[254,461, +∞)
	0.006	[0, 5,157)	[5,157, 11,889)	Dominance	[11,889, +∞)
Acceptability of screening for LTBI/TB	0.35	[0, 6,094)	[6,094, 60,378)	Dominance	[60,378, +∞)
	0.85	[0, 6,094)	[6,094, 25,180)	Dominance	[25,180, +∞)
Sensitivity of CXR	0.59	[0, 5,589)	[5,589, 34,577)	Dominance	[34,577, +∞)
	0.9	[0, 7,155)	[7,155, 7,613)	[7,613, 26,410)	[26,410, +∞)
Specificity of CXR	0.52	[0, 6,099)	[6,099, 31,526)	Dominance	[31,526, +∞)
	0.9	[0, 4,933)	[4,933, 10,165)	[10,165, 24,043)	[24,043, +∞)

Under the WTP threshold of US$50,000 per QALY gained, the base-case results were robust to all one-way sensitivity analyses except two parameters (Table 5 and S1 Fig). LTBI/TB screening was more cost-effective than TB screening (Xpert) when the probability of annual LTBI reactivation was greater than 0.155% and when its screening acceptability was greater than 38%. In two-way sensitivity analysis, the probability of LTBI/TB screening to be cost-effective increased with a higher probability of annual LTBI reactivation and its screening acceptability. Compared with TB screening (Xpert), TB screening (CXR) was no longer dominated under certain WTP thresholds when the sensitivity of CXR was greater than 0.83, the specificity of CXR was greater than 0.82, or the sensitivity of Xpert for smear negative TB was less than 0.51 and LTBI/TB screening was not available.

### Probabilistic sensitivity analysis

**Fig 3:**
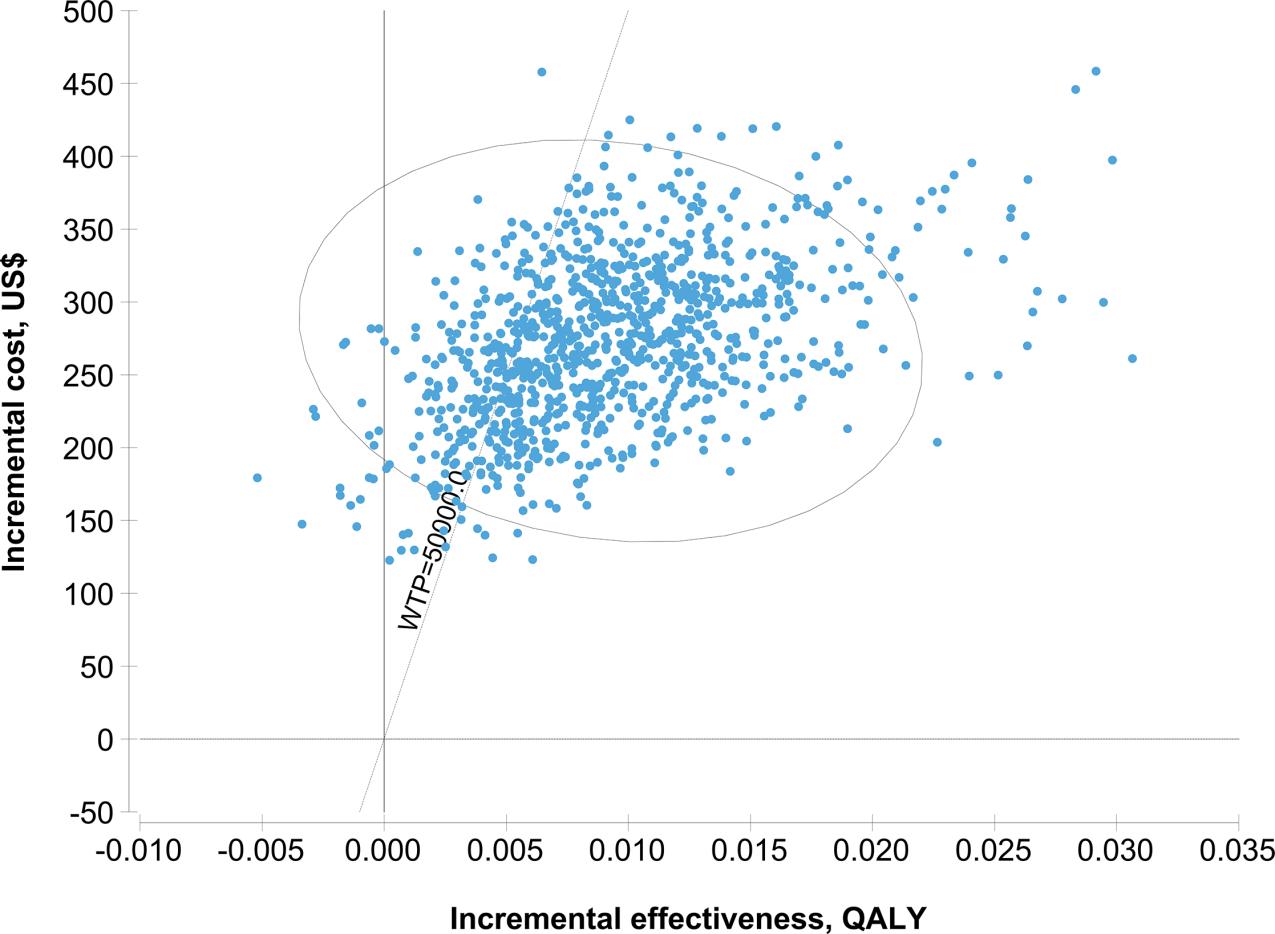
Incremental cost-effectiveness ratio of LTBI/TB screening vs. TB screening (Xpert) in 1,000 iterations of Monte Carlo simulation. The ellipse represents 95% confidence points. The diagonal line represents ICER at a WTP threshold of US$50,000/QALY. Points to the right of the diagonal line represent the iterations where LTBI/TB screening to be cost-effective.

**Fig 4:**
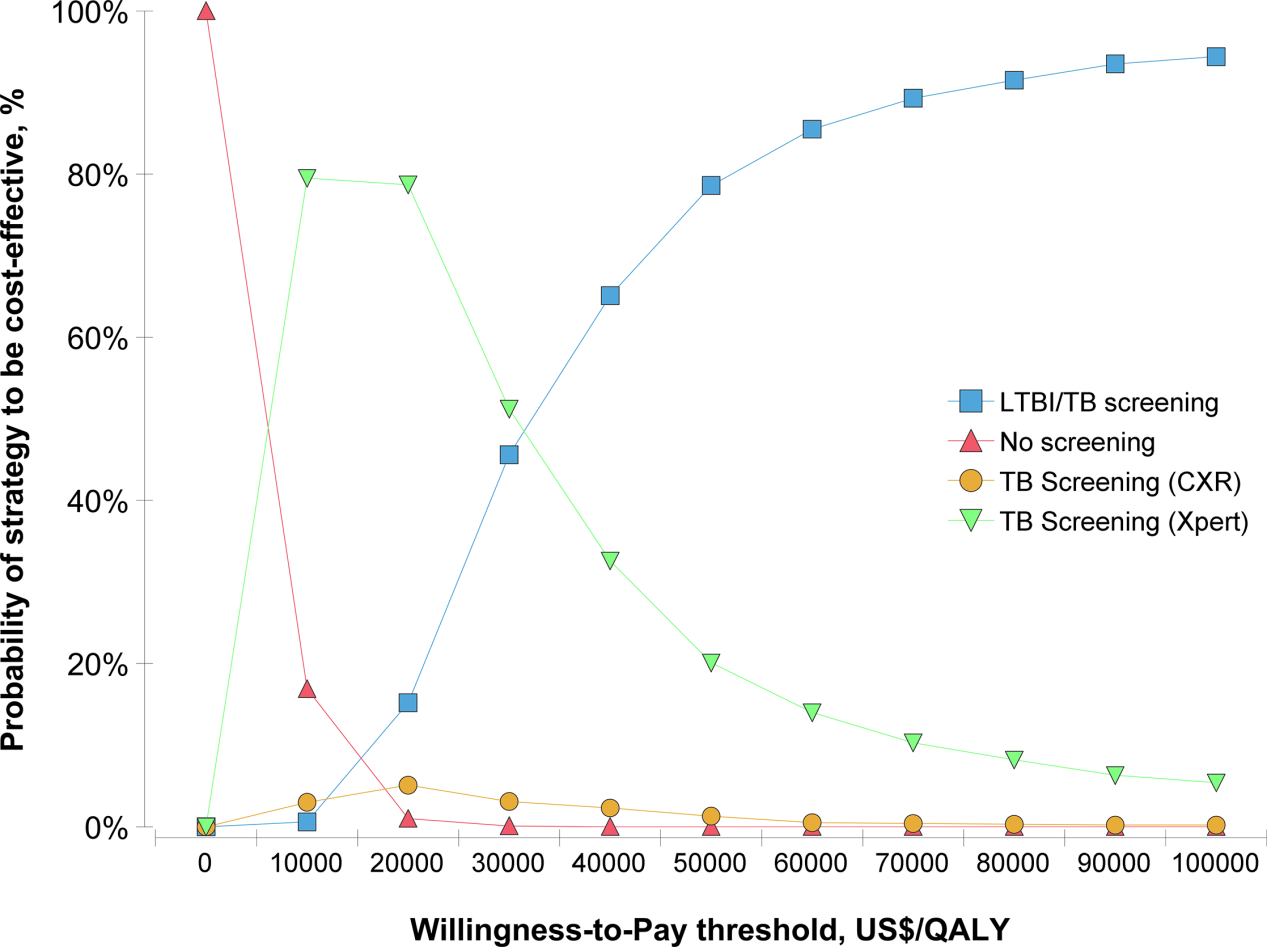
Cost-effectiveness acceptability curve for four strategies in Monte Carlo simulation.

In the 1,000 iterations of Monte Carlo simulation generated by probabilistic sensitivity analysis (S2 Table), no screening remained the most cost-saving (Median = US$120, 2.5%~97.5% quantile: 90~158) and LTBI/TB screening remained the most effective (Median = 11.1803 QALYs, 2.5%~97.5% quantile: 11.1233~11.2288). Using the WTP threshold of US$50,000 per QALY gained, LTBI/TB screening was more likely to be cost-effective compared with TB screening (Xpert) (Median of the ICER = US$25,973 per QALY gained, 2.5%~97.5% quantile: 12,405~66,629)(Fig 3 and S2 Table); the probability of no screening, TB screening (CXR), TB screening (Xpert), and LTBI/TB screening to be cost-effective were respectively 0, 1.3%, 20.1% and 78.6% (Fig 4).

## Discussion

In this study, we evaluated the cost-effectiveness of three screening strategies compared with the current no screening strategy in the elderly at admission to RCHEs. Although no screening offered the greatest cost-saving, LTBI/TB screening was the most effective strategy with highest LYs and QALYs gained and more likely to be cost-effective under the WTP threshold of US$ 50000 per QALY gained. It roughly indicates the additional actions for preventing LTBI reactivation are worthwhile. The sensitivity analyses demonstrated the robustness of result and the fact that probability of LTBI reactivation and screening acceptability influenced optimal strategy selection switching from LTBI/TB screening to TB screening (Xpert). To our knowledge, this study is among the first to evaluate the screening strategies for LTBI and TB simultaneously by new diagnostic tools in the elderly population. More useful parameters in screening algorithms, such as the symptomatic presentation and acceptability, were initially integrated into the model. In addition, a dynamic range of the WTP thresholds was adopted in decision analytic process from the health service provider perspective. The key findings would bridge the research gap and inform the TB program in Hong Kong and similar areas.

Four previous cost-effectiveness studies of TB have been reported to compare screening strategies with no screening in the elderly [31–33, 72]. Two of them demonstrated that TST screening was more cost-effective than no screening and CXR screening in long-term care facilities for the elderly in Canada [31, 33]. The risk of LTBI reactivation was also identified as an influential variable [33]. In our model design, IGRAs replaced TST in LTBI/TB screening strategy given its higher specificity and strengths. Factors such as separate test-reading visit, potential boosting of response ascribed to immune-compromised, and cross-reactivity with the BCG may affect the field application of TST especially in the elderly [15]. On the other hand, the annual risk of LTBI reactivation increased with age, e.g. from 0.20% for the 55–64 years group to 0.52% for the 65–74 years group in Hong Kong [12]. The cut-off of 0.155% for strategy-switching in our study indicates LTBI/TB screening strategy is more likely to be cost-effective in areas where the majority of elderly TB cases are developed by reactivation [15]. Also, it can be more cost-effective by targeting subpopulation with a higher risk of LTBI reactivation, such as those with TB history or high-risk medical conditions [33].

The other two studies in Japan BCG-vaccinated elderly and nursing homes demonstrated that QFT-GIT screening was the most cost-effective among screening strategies by TST, CXR and other products of IGRAs [32, 72]. The sensitivity of IGRA, prevalence of LTBI and TB, and BCG rate in TST screening were the influential variables [32, 72]. The uncertainty of these variables were not significantly observed in our study because of the higher prevalence rates in Hong Kong RCHEs and the exclusion of TST. In addition, the role of CXR alone seemed to be little in the Japan study probably due to the higher costs caused by mass screening strategy [32]. From the WHO guidelines, initial symptoms screening is recommended for a target high-risk group before CXR or Xpert screening [18]. The symptoms should be well defined and may vary in specific groups. One study in China demonstrated that screening by cough in any duration with or without hemoptysis can find additional 43% of elderly TB patients compared with cough more than 3 weeks [48]. In an attempt to balance the diagnostic yield and the feasibility of screening, we adopted a higher index of TB suspicion (i.e. cough in any duration, hemoptysis, and/or weight loss) in TB screening strategies. By comparison, TB screening (CXR) in symptomatic elderly resulted in a lower ICER in our study and can be conditionally considered as an alternative in places where the Xpert is unavailable.

The acceptability of screening could be a key factor affecting the field application. Two previous studies examined that the acceptability of screening for TB in elderly people living in institutions ranged from 72% to 95% [18], while evidence is still lacking in screening and treating for LTBI. Despite more than 70% of the elderly screened in Hong Kong RCHEs studies, preventive therapy was not provided at that time and might raise concerns in its applications [7, 8]. In Hong Kong, people older than 35 year-old are not recommended for LTBI screening given the understanding that serious side effects in preventive therapy increase with age [10]. Among them, the risk of hepatotoxicity and mortality in the elderly is mainly concerned. However, recent studies demonstrated abnormal liver function absent of monitoring and quick response had more impacts than age on the development of isoniazid-induced hepatotoxicity [50, 73]. In a systematic review of the age-related risk of hepatotoxicity by treating LTBI, the overall rates of hepatotoxicity were low; associated hospitalization or mortality was extremely uncommon [50]. In response, we provided health education, liver function and hepatitis test before treatment, regular following-up clinical and biochemical monitoring, as well as hospitalization once serious side effects were found to reduce the potential harm. We also adopted a wider range of the acceptability rate of LTBI screening for the sensitivity analysis. The result implies more attention should be paid if the acceptability rate is expected to be lower than around 40%.

In addition to the comparison among strategies, the decision making also depends on how much the health service providers are willing to pay. To date, the recommendations for WTP thresholds are diverse in different countries and circumstances: e.g. 50,000 to 10,000 US$ per QALY in the USA, £20,000 to £30,000 per QALY in the UK, one to three times the per capita annual income from the WHO recommendation, and other higher thresholds considering the inflation [74, 75]. Given few recommendations in Hong Kong, we adopted 50,000 US$ per QALY gained in line with the majority of cost-effectiveness studies. We also provided the ranges of WTP thresholds for strategies to be optimal and made decision making by multiple thresholds possible [75]. Strategy selection can be optimized and updated given the available resources and alternative uses in the local TB program [75].

In the Markov model, a dynamic transmission of TB was not considered. We adopted a fixed ARI to simulate the new transmission in the RCHEs, which was not sensitive to the changing number of infectious cases. In reality, the effect of dynamic transmission could be minimized by effective infection control measures, early detection, and appropriate treatment. In the Markov states, we assumed a high prevalence of LTBI and TB based on previous studies using TST as the initial screening test between 1996 and 2006. There might be a risk of overestimation given the limitations of TST and a decline of recent TB notification rates in the elderly. Following-up evaluation by IGRAs and an update of TB epidemiological survey would improve the model accuracy. In the model structure and parameters, the diagnostic algorithm for smear-negative patients was simplified as the combination of CXR with or without GFT-GIT, sputum smear and culture test, diagnostic antibiotic trial, and clinical judgment; response to complex adverse events in treatment was limited to side effects such as hepatotoxicity given its severity and representativeness. The costs in this study might be underestimated as certain expenditure for additional radiologic tests, bronchoscopy, response to complications, drug intolerance, and other minor side effects were not included due to the variability in their applications. In addition to the clinical performance from systematic reviews, some parameters were collected from studies in similar settings. They were therefore examined over a wide range of values in the sensitivity analyses. The proportion of symptoms and the willingness to approach healthcare facilities, the acceptability of LTBI screening and preventive therapy, the accuracy of diagnostic tools, as well as the utility weight of health status related to TB warrant more research to validate our results.

## Conclusions

This study informed the health policymakers that screening for latent and active TB infection in the elderly at admission to Hong Kong RCHEs is highly effective and cost-effective, based on the WTP threshold of 50,000 US$ per QALY gained and a higher probability of LTBI reactivation and screening acceptability. In intermediate TB burden areas where health service providers work hard to find breakthrough strategies to further bring down TB endemic in the elderly population, our findings provide an alternative solution that warrants further research.

## Supporting information

S1 Table Probabilistic distribution of parameters used in the probabilistic sensitivity analysis. (DOCX)

S2 Table Results of probabilistic sensitivity analysis. (DOCX)

S1 Fig Results of one-way and two-way sensitivity analysis. (TIF)
