# Supplementary material for: Screening for latent and active tuberculosis infection in the elderly at admission to residential care homes: A cost-effectiveness analysis in an intermediate disease burden area
Source: PLoS One. 2018 Jan 2;13(1):e0189531. doi: 10.1371/journal.pone.0189531 (PMC5749681; doi:10.1371/journal.pone.0189531)
Supplement: S1 Table — (DOCX) [file pone.0189531.s001.docx]

**S1 Table. Probabilistic distribution of parameters used in the probabilistic sensitivity analysis.**

| Model parameters | Baseline value | Distribution | Distribution parameters | | | |
| --- | --- | --- | --- | --- | --- | --- |
|  |  |  | α | β | µ | σ |
| Prevalence of LTBI | 0.57 | Beta | 55.88 | 42.16 |  |  |
| Prevalence of TB | 0.012 | Beta | 7.03 | 578.46 |  |  |
| Annual risk of TB infection | 0.013 | Beta | 41.7 | 3166.05 |  |  |
| Probability of annual LTBI reactivation | 0.0025 | Beta | 6.23 | 2487.52 |  |  |
| Probability of TB relapse after treatment | 0.012 | Beta | 35.57 | 2928.43 |  |  |
| Probability of successful treatment among TB patients | 0.766 | Beta | 343.25 | 104.86 |  |  |
| Probability of TB death in treated patients |  |  |  |  |  |  |
| age 65-69 years old | 0.028 | Beta | 7.62 | 264.54 |  |  |
| age 70-74 years old | 0.069 | Beta | 44.32 | 598.07 |  |  |
| age 75-79 years old | 0.068 | Beta | 43.1 | 590.66 |  |  |
| age 80-84 years old | 0.084 | Beta | 64.63 | 704.81 |  |  |
| Probability of death by other causes |  |  |  |  |  |  |
| age 65-69 years old | 0.009 | Beta | 891.9 | 98208.1 |  |  |
| age 70-74 years old | 0.017 | Beta | 1136.35 | 65707.7 |  |  |
| age 75-79 years old | 0.027 | Beta | 2837.27 | 102247 |  |  |
| age 80-84 years old | 0.047 | Beta | 8420.71 | 170743 |  |  |
| Probability of TB death in untreated smear positive patients | 0.113 | Beta | 28.32 | 222.26 |  |  |
| Probability of TB death in untreated smear negative patients | 0.022 | Beta | 18.93 | 841.71 |  |  |
| Proportion of smear positive in annual registered TB patients | 0.376 | Beta | 882.19 | 1464.05 |  |  |
| Probability of cough in any duration, hemoptysis and/or weight loss in elderly population | 0.2 | Beta | 12.8 | 51.2 |  |  |
| Probability of cough ≥3 weeks and/or hemoptysis in elderly population | 0.0327 | Beta | 1034.32 | 30596.4 |  |  |
| Probability of cough in any duration, hemoptysis and/or weight loss in elderly TB patients | 0.6 | Beta | 57.6 | 38.4 |  |  |
| Probability of cough ≥3 weeks and/or hemoptysis in elderly TB patients | 0.36 | Beta | 33.18 | 58.98 |  |  |
| Probability of willingness to consult for clinical services when cough ≥3 weeks and/or hemoptysis | 0.7 | Beta | 58.8 | 25.2 |  |  |
| Sensitivity of CXR | 0.7 | Beta | 18.15 | 7.78 |  |  |
| Specificity of CXR | 0.6 | Beta | 57.6 | 38.4 |  |  |
| Sensitivity of Xpert for smear negative TB | 0.72 | Beta | 29.62 | 11.52 |  |  |
| Sensitivity of QFT-GIT | 0.84 | Beta | 1128.96 | 215.04 |  |  |
| Acceptability of screening for TB | 0.83 | Beta | 48.85 | 9.59 |  |  |
| Acceptability of screening for LTBI/TB | 0.6 | Beta | 14.4 | 9.6 |  |  |
| Adherence rate of IPT | 0.8 | Beta | 51.2 | 12.8 |  |  |
| Efficacy of IPT | 0.85 | Beta | 43.35 | 7.65 |  |  |
| Probability of isoniazid-induced hepatotoxicity | 0.017 | Beta | 7.89 | 456.3 |  |  |
| Probability of hepatotoxicity in TB treatment | 0.086 | Beta | 270.4 | 2873.76 |  |  |
| The average days of hospitalization | 15 | Log-normal |  |  | 2.7 | 0.14 |
| Cost of Xpert test | 128 | Log-normal |  |  | 4.85 | 0.1 |
| Cost of QFT-GIT test | 70 | Log-normal |  |  | 4.24 | 0.1 |
| Cost of TB hospitalizations per day | 600 | Log-normal |  |  | 6.39 | 0.1 |
| Cost of diagnosis antibiotic trial | 340 | Log-normal |  |  | 5.83 | 0.08 |
| Average physician income per hour | 72 | Log-normal |  |  | 4.27 | 0.1 |
| Average nurse income per hour | 40 | Log-normal |  |  | 3.68 | 0.1 |
| Utility of treated active TB disease | 0.85 | Beta | 43.35 | 7.65 |  |  |
| Utility of untreated active TB disease | 0.7 | Beta | 29.99 | 12.86 |  |  |
| Utility of drug-related hepatotoxicity | 0.8 | Beta | 142.22 | 35.56 |  |  |
