# Supplementary material for: Screening for latent and active tuberculosis infection in the elderly at admission to residential care homes: A cost-effectiveness analysis in an intermediate disease burden area
Source: PLoS One. 2018 Jan 2;13(1):e0189531. doi: 10.1371/journal.pone.0189531 (PMC5749681; doi:10.1371/journal.pone.0189531)
Supplement: S2 Table — (DOCX) [file pone.0189531.s002.docx]

**S2 Table. Results of probabilistic sensitivity analysis**

| **Strategy** | **Median (2.5%~97.5% quantile) of cost (US$) per person** | **Median (2.5%~97.5% quantile) of Incremental cost (US$)** | **Median (2.5%~97.5% quantile) of QALYs accrued per person** | **Median (2.5%~97.5% quantile) of incremental QALYs gained** | **Median (2.5%~97.5% quantile) of ICER** | **Median (2.5%~97.5% quantile) of ICER** |
| --- | --- | --- | --- | --- | --- | --- |
| No screening | 120 (90~158) | Reference | 11.1638 (11.0999~11.2180) | Reference | Reference | - |
| TB Screening (Xpert) | 161 (126~205) | 40 (25~59) | 11.1698 (11.1095~11.2241) | 0.0066 (0.0025~0.0142) | 6,187 (2,682~16,934) | Reference |
| TB screening (CXR) | 166 (130~214) | 46 (29~70) | 11.1686 (11.1074~11.2233) | 0.0050 (0.0016~0.0111) | 9,200 (3,699~27,363) | -2,757  (-52,807~46,405) |
| LTBI/TB screening | 436 (324~558) | 314 (205~424) | 11.1803 (11.1233~11.2288) | 0.0152 (0.0068~0.0294) | 20,171 (11,252~41,275) | 25,973  (12,405~66,629) |
