# Supplementary figures and images for: Screening for latent and active tuberculosis infection in the elderly at admission to residential care homes: A cost-effectiveness analysis in an intermediate disease burden area
Source: PLoS One. 2018 Jan 2;13(1):e0189531. doi: 10.1371/journal.pone.0189531 (PMC5749681; doi:10.1371/journal.pone.0189531)

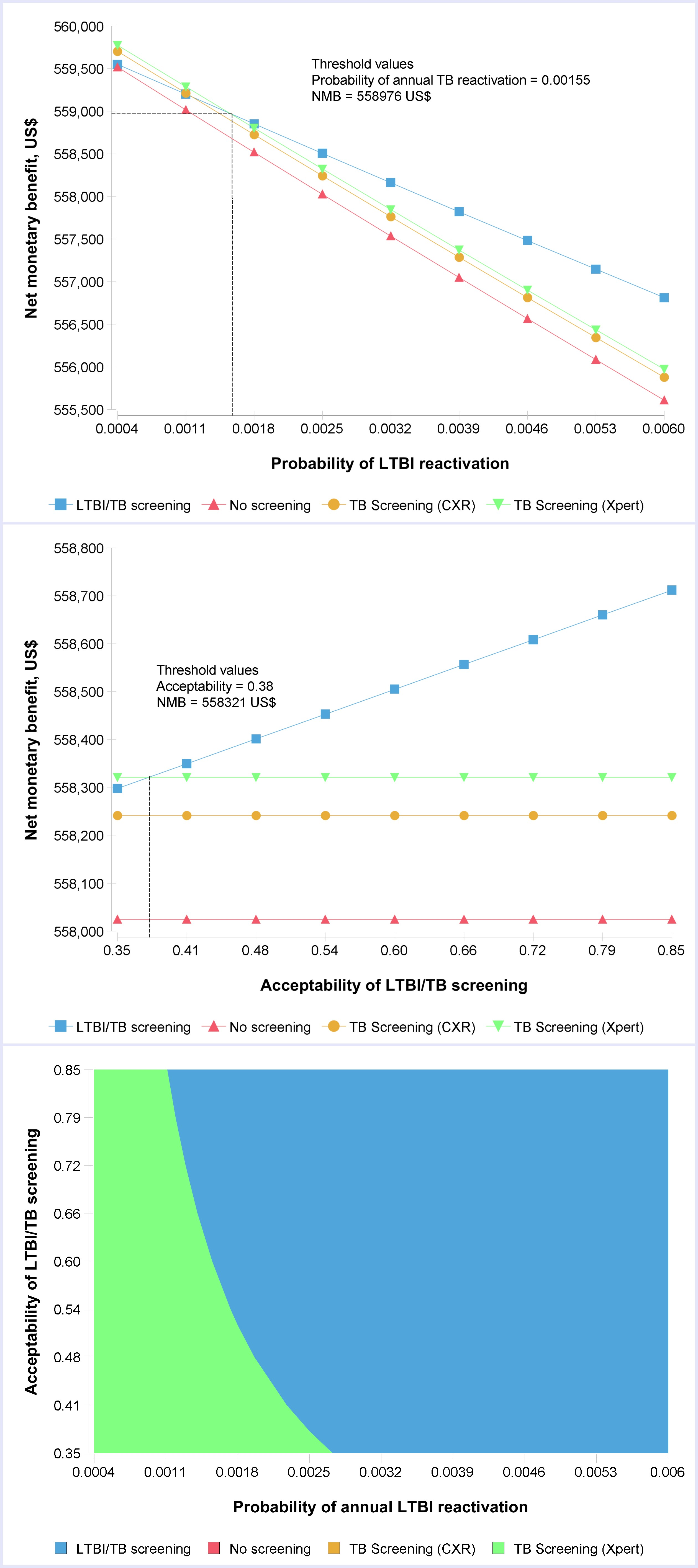

Supplement: S1 Fig — (TIF) [file pone.0189531.s003.tif]
